# Three-year outcomes with left ventricular assist devices in country with restricted heart transplantation

**DOI:** 10.1186/1749-8090-10-S1-A32

**Published:** 2015-12-16

**Authors:** Assel Medressova, Serik Bekbossynov, Muradym Murzagaliyev, Saltanat Dzhetybayeva, Saltanat Andossova, Makhabbat Bekbossynova, Yuriy Pya

**Affiliations:** 1 National Research Center for Cardiac Surgery, Astana, Kazakhstan

## Background/Introduction

As a consequence of limited donor availability, there has been a growing interest for alternative strategies, such as left ventricular assist devices (LVAD) as either a bridge to transplantation (BTT) or as destination therapy (DT) for the treatment of the advanced heart failure. The heart transplant program in Kazakhstan is in a nascent stage and therefore patients that are determined to be BTT are expected to have an extended duration of LVAD support.

## Aims/Objectives

The objective of this study was to determine outcomes of patients with left ventricular assist devices in country with restricted transplantation.

## Method

We analyzed outcomes for 135 patients between November 2011 and November 2014 (mean age=50,5 ± 13,5 years old; Heart Mate II=95 (70,4%), HeartWare=40 (29,6%)). The median duration of support is 474 ± 329,4 days.

## Results

In 75 patients (55,6%) the LVAD is used as a BTT and in 60 (44,4%) as a DT, but only 3 of 135 LVAD patients were transplanted. Before 30 days after implantation of LVAD right ventricular failure (n = 20, 14,8%), renal failure (n = 19, 14,1%) and bleeding (requiring reoperation = 10, 7,4%, requiring transfusion of packed red blood cells ≥4U = 23, 17,04%) were the most common adverse events. After 30 days driveline infections (n = 46, 34,1%) and strokes (n = 33, 24,44%) were the most common complications. Cumulative survival rates at 1, 6 months, 1, 2 and 3 years after LVAD implantation is 93%, 86%, 77%, 62% and 51% accordingly. Older age and more acute INTERMACS profiles were related to reduced survival.

## Discussion/Conclusion

The Center's experience shows that LVADs can be implanted as an alternative to heart transplantation with the outcomes that are comparable to those in existing world centers of excellence.

